# PD73 Oncology Drugs Are Underrepresented In The Incorporation Process In Brazil

**DOI:** 10.1017/S0266462325102043

**Published:** 2025-12-29

**Authors:** Elene Paltrinieri Nardi, Ricardo Rodrigues Teixeira, Daniela Melo

## Abstract

**Introduction:**

Since 2011, the National Committee for Health Technology Incorporation (CONITEC) has advised the Brazilian Ministry of Health on incorporating or disinvesting health technologies. In oncology, there is no comprehensive list of the drugs available in the Unified Health System (SUS) because financing relies on procedure reimbursements to clinics and hospitals. This study evaluated whether oncology drugs marketed in Brazil have been assessed by CONITEC.

**Methods:**

We compiled a list of oncology drugs approved in Brazil between 2010 and 2020. Regulatory approval data and indications were obtained from the Brazilian Health Regulatory Agency (ANVISA) website. For each drug, all labeled indications were extracted. We then analyzed whether the drug-indication pairs had been evaluated by CONITEC by December 2022 and identified the entities requesting these evaluations.

**Results:**

A total of 134 drug-indication pairs were identified. CONITEC conducted technology appraisals for 28 pairs (20.9%), while 106 pairs (79.1%) remained unevaluated as of 2022. Among the 28 assessed pairs, the majority received negative recommendations for incorporation into the SUS (n=16; 57.1%). The Ministry of Health was the primary initiator of these evaluations, accounting for 17 of the 33 requests (51.5%), followed by medical societies (n=6; 18.2%), pharmaceutical companies (n=5; 15.1%), and others (n=5; 15.1%).

**Conclusions:**

Most oncology drugs in Brazil have not been evaluated by CONITEC, and most assessed drugs were not recommended for incorporation. This may be a reflection of the oncology care financing model, potentially exacerbating funding and economic disparities within the public health system.

